# Preclass video nanolearning or microlearning in blended medical education

**DOI:** 10.3389/fmed.2025.1639475

**Published:** 2025-10-07

**Authors:** Cheng-Maw Ho, Chi-Chuan Yeh, Jann-Yuan Wang, Rey-Heng Hu, Po-Huang Lee

**Affiliations:** ^1^Department of Surgery, National Taiwan University Hospital, Taipei, Taiwan; ^2^School of Medicine, College of Medicine, National Taiwan University, Taipei, Taiwan; ^3^Department of Internal Medicine, National Taiwan University Hospital, Taipei, Taiwan; ^4^Center of Faculty Development and Curriculum Integration, College of Medicine, National Taiwan University Hospital, Taipei, Taiwan; ^5^Department of Surgery, E-Da Hospital, I-Shou University, Kaohsiung, Taiwan

**Keywords:** video length, microlearning, nanolearning, blended medical education, mixed-methods design

## Abstract

**Background:**

Short educational videos, including microlearning (multi-concept) and nanolearning (single-concept), are increasingly used in blended medical education.

**Objective:**

This study examined medical students’ preferences, learning behaviors, and outcomes when engaging with nanolearning versus amalgamated microlearning videos.

**Methods:**

A convergent parallel mixed-methods design was used. Fifth-year medical students accessed core concept videos in both formats and completed a questionnaire on format preference and learning experience. Data included questionnaire responses, online activity logs, and assessment scores, analyzed using statistical tests and thematic analysis.

**Results:**

Of 156 students, 140 responded; 79 preferred microlearning, 18 preferred nanolearning, and 43 had no strong preference. While video engagement time did not differ, nanolearning-preferring students more often completed individual concepts. The “either” group reported higher satisfaction, and the microlearning group performed better on essay assessments. Regardless of format, students found the short videos convenient and effective.

**Conclusion:**

Students valued both nanolearning and microlearning formats for pre-class preparation. Incorporating short, flexible video formats may enhance engagement and learning in medical education.

## Introduction

Online video-based learning has been incorporated into medical education due to its flexible nature, eliminating geographical restrictions and scheduling limitations (1, 2). By integrating online video learning into the framework of blended learning, its advantages are amplified (3). It enables medical students to acquire and enhance their foundational knowledge of a subject before attending face-to-face classes, facilitating a more comprehensive learning experience (1). As social media popularizes the use of short videos to convey messages, these videos have received tremendous feedback from the general population (4, 5). In the realm of on-the-job training and learning, there is a growing interest in short, several-minute-long videos that focus on a few key points or even just one (4, 6). This approach, known as micro or nanolearning, involves lessons shorter than 15 min and can utilize various content formats such as videos, texts, images, tests, and games, among others (4, 7). Evidence indicates that microlearning in health professions education leads to learner satisfaction, improved knowledge and attitudes (8, 9). These features and advantages highlight the immense potential for its wide application in blended medical education (10).

Microlearning is an emerging pedagogy that enables students and clinicians to engage in short, focused, asynchronous and just-in-time learning (9). The purpose of microlearning is not simply to divide content into multiple smaller lessons, but to pick out and condense the most crucial content so that it can all be consumed in one self-contained bite-sized lesson (11). The single piece of information or learning objective that is delivered can be called an information snippet, nugget or microunit (9). The acquisition of meaningful medical knowledge or skills often involves integrating smaller background information pieces. Therefore, microlearning could be dissected into multiple parts of nanolearning, representing an even smaller unit (12).

Nanolearning is characterized as “quick hits” of information (6) by offering concise learning capsules that synthesize maximum useful information, typically presenting one key point per capsule (13). While both involve breaking down information into small, easily digestible pieces, microlearning typically refers to slightly longer pieces of content, usually between 5 and 15 min in length (14). Nanolearning, on the other hand, refers to even shorter pieces of content, often no longer than 2 minutes (14). There is no strict definition of time-frame duration to distinguish nanoleaning from microlearning in academic community yet. Nonetheless, in contrast to full-length pre-recorded lecture videos, the advantage of micro- or nanolearning lies in its succinct nature (1, 7, 13). Although these terms are actually highly contentious and ill-defined in the educational media community, the platform of online video-based learning in blended medical education is potentially a great area to adopt these strategies (10). It is yet to be established which knowledge and skills are best suited for learning in focused snippets (9). This study presents pilot data reflecting students’ preferences between nanolearning—defined as one-minute videos focused on a single concept—and microlearning, characterized by a six-minute video covering all six concepts.

### Theoretical framework

The increasing integration of short educational videos into blended medical education has highlighted the need to better understand how nanolearning and microlearning formats influence learning outcomes and student engagement. Theo Hug, a pioneer in the conceptual development of microlearning, emphasized key characteristics including brevity, a singular learning objective or “knowledge nugget,” and on-demand accessibility across various media formats (15). His work, along with that of Thillainadesan et al., defined microlearning as content that is short, focused on a single outcome, and available anytime, anywhere—characteristics that make it particularly suitable for the demands of modern health professions education (9, 15).

In a recent scoping review, microlearning was shown to have positive impacts on knowledge acquisition, skill performance, knowledge retention, study habits, and collaborative engagement among health professions students (8, 9). These findings support its role as a promising educational strategy in both procedural and cognitive domains. However, while microlearning is often described as short, the specific segmentation approach—whether through amalgamated short videos or modular nanolearning units—remains an area of active investigation.

Our research is further grounded in cognitive load theory and multimedia learning theory, which emphasize the importance of segmenting content to reduce extraneous load and enhance learning efficiency (1, 16–19). Segmenting, or “chunking,” information can be achieved through either internal breaks (“click-forward” pauses) or by dividing content into shorter, standalone videos (20). Prior studies have demonstrated the educational value of both strategies, particularly in maintaining learner engagement and promoting video-based learning (20–23). However, it remains unclear which method—amalgam microlearning or nanolearning video segmentation—is more effective in practice.

Our previous work applied these theoretical insights in the development of blended precision medical education, demonstrating that preclass short video-based learning can help balance cognitive load while maintaining learner engagement (1). In earlier iterations, we produced microlearning videos (10–12 min) covering six key concepts within a single topic, such as acute liver failure (1, 24). In response to student feedback requesting even shorter segments, we later created nanolearning videos (1–2 min each) for individual concepts (10, 25). However, subsequent student feedback revealed divergent preferences, prompting us to upload both versions—the full microlearning video and the segmented nanolearning series—allowing students to choose their preferred format.

This evolving implementation set the stage for our current inquiry. Given the differences in content structure and presentation, we hypothesized that students’ choice of video format (amalgam, nanolearning, or either fine) would be associated with distinct patterns of engagement, learning outcomes, and self-reported learning experiences. Therefore, our study aims to investigate:

1. What are medical students’ preferences and perceived behaviors when comparing educational nanolearning videos to an amalgam microlearning video?
2. What student-reported experiences and learning outcomes are associated with these preferences?

By aligning these questions with established theories and empirical evidence, this study seeks to generate insights that can inform adaptive teaching strategies and support more personalized learning pathways within blended medical education.

## Methods

### Participants

Most participants were 23–24 years old (*n* = 124 for age 23, *n* = 12 for age 24, *n* = 2 for age 22, *n* = 1 for age 25, and *n* = 1 for age 26). The study focused on acute liver failure as a section of a mandatory core course of surgery for the fifth-year medical students. Each course section consisted of a 1-h class with 26 medical students enrolled in each round. A round in the surgery program spans 6 weeks, and a total of six rounds (learning group 1–6) are conducted in 1 year. Students were allocated to one of the six learning groups by the medical school. Between September 2022 and May 2023, a total of 156 fifth-year medical students took the course and were invited to participate in the questionnaire. All procedures of the study were carried out in accordance with the Declaration of Helsinki and the Institutional Review Board of National Taiwan University Hospital approved this study as an exempt protocol for multiple academic years (201809078 W and 202006048 W). Participation in the questionnaire was considered to imply written consent.

### Research design

**Figure 1:**
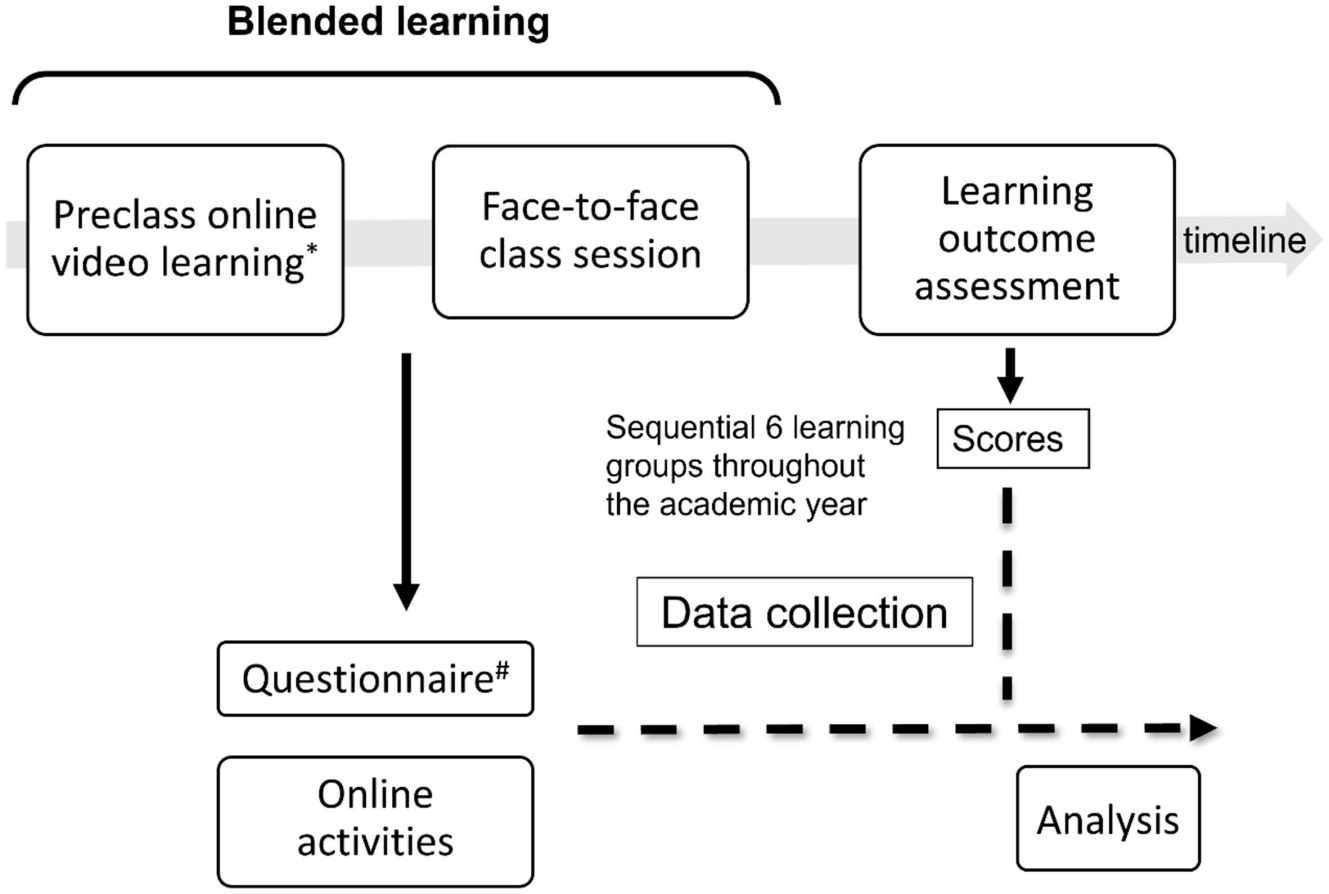
Course design, data collection process, and study analysis. Two types of videos were available online for students to watch freely before the face-to-face class session. One type included six individual, very short (one-minute long) videos, each focusing on a specific concept (nanolearning). The other type was an amalgam microlearning video covering six concepts in a six-minute long video (microlearning). A total of seven video clips were available online, and students provided their feedback on their preferred video type (amalgam, nanolearning, or either fine). Data were collected after the completion of the surgery course for each learning group. There were six learning groups sequentially in an academic year. Note that before the face-to-face class session for a learning group, both qualitative free comments and quantitative data from the questionnaire were collected. ^*^ Students can choose freely to watch the amalgam microlearning video or individual nanolearning videos. ^#^ Including students’ preferences regarding the preclass video types (amalgam, nanolearning, or either fine).

To provide a more comprehensive understanding of the research questions, the study employed a convergent parallel mixed-methods design, using quantitative data from questionnaires, online records, and assessment scores for statistical analysis, and qualitative data from open-ended questionnaire responses for thematic analysis. A cross-sectional questionnaire (see Figure 1) involved both quantitative and qualitative components, namely scale-based questions and open-ended sections where participants could provide free comments (10, 25, 26). Dataset derived from the academic year (September 2022 to May 2023) was analyzed exclusively in this study.

### Definition

In broad terms, nanolearning is a smaller unit scale of microlearning, and nanolearning materials can be segments of a microlearning topic. In this study, we defined a nanolearning video as a very short video lasting 1 minute, featuring a specific core concept (C) per video. The amalgam microlearning video was a collection of six core concepts (C1 to C6 in consecutive order) within one video, lasting 6 minutes, and presenting the topics of acute liver failure as a typical microlearning video.

### Course design

Cheng-Maw Ho (HCM) was assigned by the curriculum development committee to develop the course section on acute liver failure. Due to the limited official time allocated to this topic (1 h per round), the teacher had to employ strategies to manage time constraints and address individual learning gaps as effectively as possible. HCM incorporated six threshold concepts of acute liver failure (details previously (1, 24)), based on educational goals, into the course design and practice (10, 25). A blended course section on acute liver failure has been implemented since 2018.

### Course practice

The course proceeded in two stages: preclass online video learning and face-to-face classroom instruction. The online video portion of the course was designed based on the coherence principle (excluding extraneous content) and the segmenting principle (allowing content to be presented at the learner’s pace instead of as a continuous video) (18, 19). Six video clips, each lasting less than 2 min, were created to minimize cognitive load (16, 17). Each video had an eye-catching title and a summary of the core concept (footnotes of Table 1) (10, 25). Besides six independent videos were uploaded, another amalgam version of these clips combined was uploaded to the same intranet webpage (27). Students had the freedom to choose which videos to watch and learn from. The websites included updated review literature for reference reading, supplementing further self-learning for students who wished to delve deeper into the topic (27).

**Table 1:** Characteristics of participants and their responses after preclass online video learning, stratified by preclass video format preferences.

Variables	All	Amalgam	Nanolearning	Either fine	*P*	
*n*	140	79	18	43	Amalgam vs. Nanolearning	All 3
Male	103	54	14	35	0.572	0.292
Learning group					0.880	0.812
1	23	13	4	6		
2	25	15	3	7		
3	24	13	4	7		
4	22	12	1	9		
5	23	16	2	5		
6	23	10	4	9		
Level of satisfaction	138				0.527	0.055*
Very satisfied	64	31	8	25		
Satisfied	62	40	7	15		
Neither satisfied nor dissatisfied	12	8	3	1		
Dissatisfied	0	0	0	0		
Very dissatisfied	0	0	0	0		
Completion percentages of preclass video viewing (Median, IQR)	140	79	18	43		
C1	80.5 (0–100)	0 (0–100)	99 (93.25–100)	94 (0–100)	0.006	0.019
C2	1.0 (0–100)	0 (0–98)	100 (96.5–100)	2 (0–100)	0.001	0.006
C3	0 (0–100)	0 (0–100)	100 (96.0–100)	0 (0–100)	<0.001	0.002
C4	0 (0–100)	0 (0–100)	99 (96–100)	0 (0–100)	0.006	0.020
C5	0 (0–99)	0 (0–99)	99 (99–99)	0 (0–100)	<0.001	0.002
C6	0 (0–100)	0 (0–100)	100 (97.75–100)	24 (0–100)	0.001	0.003
Amalgam C	100 (0–100)	100 (89–100)	0 (0–100)	93 (0–100)	<0.001	<0.001
Class format preference					>0.999	0.918
Face-to-face	45	24	5	16		
Online	63	37	9	17		
Both	32	18	4	10		
Providing comments	87	52	10	25	0.427	0.565
Total online time (min)	25.9 (15.4–47.5)	26.0 (15.8–48.6)	32.6 (15.1–45.2)	23.1 (10.7–48.9)	0.922	0.791
Webpage counts	88 (67.3–112)	87 (71–112)	102 (82–119.5)	83 (57–107)	0.254	0.346
Scores						
Essay question	2.5 (1.5–3.0)	3 (1.5–3.0)	2 (0.75–3.0)	2.5 (1.5–3.0)	0.027	0.057
Clinical case analysis	90 (90–93)	90 (90–93.8)	90 (88.5–94)	90 (90–92)	0.637	0.542

* Amalgam vs. either fine, *P* = 0.018.

C, core concept^#^; IQR, interquartile range.

# Core concepts of the ‘acute liver failure’ course (24).

C1. Acute liver failure is a systemic syndrome.

C2. The international normalized ratio of prothrombin time is a diagnostic and prognostic factor of acute liver failure.

C3. Blood urea nitrogen and phosphate are liver-related biomarkers in acute liver failure.

C4. Renal dysfunction due to hepatorenal syndrome is much less frequently observed than that due to dehydration, infection, or drug toxicity.

C5. Deterioration of hepatic encephalopathy can be reversed by correcting trigger/aggravating factors—usually infection, bleeding, or dehydration.

C6. Macrophage plays a vital role in body fluid status dynamic fluctuation during the disease course.

### Short-term outcome assessment

Two summative assessments related to the acute liver failure section, as previously described (10, 25), were objective assessments of short-term outcome. Briefly, the first assessment was a written exam, a short essay question on acute liver failure valued at 3 points. The second assessment was a clinical case-based analysis, worth up to 100 points. Clinical cases, available on the intranet (27), were selected for analysis and students were required to select a clinical case and conduct a critical analysis based on their acquired knowledge. This analysis was submitted online before the end of the surgery course. The clinical teacher (HCM) graded the students’ work after the course was completed.

### Online questionnaires

At the beginning of the surgery course, students were instructed to watch the online videos in advance. Subsequently, they were invited to respond to an online questionnaire (details provided in reference (9); Supplementary Table S1) accessible on the intranet. The questionnaire, validated in English, consisted of 12 items without sub-dimensions (10). It included a mix of question styles, including 5-point Likert-type questions. The reliability of the questionnaire had been assured previously (24). An additional question regarding video learning preferences (“I prefer the video format for learning as an amalgam one, separate items, or either fine?”) was included. The questionnaire evaluated various aspects, including the degree of agreement with concepts after pre-class online video learning compared to prior understanding (rated on a scale of 1 to 5), concepts requiring additional instruction in future classes, concepts could be deleted, concepts learned most by the student, satisfaction with the use of online videos before class, preference for class format (face-to-face, online, or both), and space for student comments or questions. Based on the questionnaire responses, the teacher developed the content for the upcoming classes in each round, aiming to address learning gaps and provide precision medical education (1).

### Online learning activities

Anonymous documentation was made of cumulative website page views and webpage visit/browsing durations for each medical student. Video completeness was automatically calculated by determining the ratio of the duration a video was played to the total video duration, excluding fast forwarding, rewinding, or skipped segments (10).

### Data collection

The data collection process is shown in Figure 1. Free comments, as a qualitative component of the questionnaire data, were collected alongside the quantitative data before the face-to-face class session for a learning group. For each round of students, an administrative teaching assistant facilitated the collection and matching of anonymous questionnaire data, demographic information, scores, and automatically captured online activity details by the system (10). Altogether, there were sequential 6 learning groups throughout the academic year.

### Qualitative data analysis

Student comments were independently coded using a descriptive thematic analysis approach by a specialist surgeon and an administrative researcher, following the guidelines by Braun and Clarke (1, 10, 25, 28). A code management policy had been established previously (10, 25), and any coding discrepancies were resolved through discussions among a team consisting of a surgeon specialist, an experienced medical education specialist, and an administrative researcher. The team met regularly to combine codes and ensure consistency. No additional meetings were scheduled after the coders agreed that thematic saturation had been reached. Based on the questionnaire responses from each round of students, the teacher in charge addressed students’ questions and adjusted the content weight of the classes (1).

At the end of the school year, a final set of identified themes was generated to represent the range of student feedback on the learning process (10). Research questions were used to collect students’ thoughts on their self-reported learning process and experiences with the course framework design, particularly their preferences regarding preclass online video learning. Each student comment might encompass several codes across different categories, including general feedback, learning experiences and queries, course design, video specifications, and miscellaneous comments.

### Quantitative data analysis

Quantitative data were summarized using means, medians, or percentages. Non-parametric tests such as the Kruskal-Wallis or Chi-square tests were employed to compare group differences. A multiple general linear regression model was utilized to identify potential factors associated with preclass video format preferences, using a backward elimination method. A two-sided *p*-value of less than 0.05 was considered statistically significant. Statistical analyses were performed using SPSS version 21.0 (SPSS, Chicago, IL, United States).

## Results

### Student characteristics, online video learning activities, and pre-class video format preference

Of the 140 fifth-year medical students (140/156, 89.7%) who participated in online video learning and completed the questionnaire on their pre-class video format preferences (Supplementary Figure S1), the majority were male (73%) and expressed satisfaction with the pre-class online video learning process (91.3%). They also provided comments (62.1%), with 56.4% preferring the amalgam microlearning video format, 12.9% preferring the nanolearning video format, and 30.7% having no specific preference (either fine) (Table 1). Participants spent a median of 25.9 min online, including time for watching the videos, and had a median number of 88.0 webpage visits, without significant differences among the groups (Table 1).

The completion rate of pre-class amalgam video viewing was higher among students who preferred the amalgam microlearning video format compared to the other groups (*p* < 0.001), while the completion rate of individual concept viewing was higher among students who preferred the nanolearning video format (*p* < 0.05 for all six concepts) (Table 1). Interestingly, in the amalgam group, 19 students watched nanolearning videos with high completion rates (>90%) and low rates (<30%) for amalgam microlearning videos. In the nanolearning group, six students watched amalgam microlearning videos with high completion rates. In the either fine group, 23 students viewed amalgam microlearning videos and 10 viewed individual videos with high completion rates. This group had high completion rates in C1 nanolearning video (94%) and amalgam microlearning video (93%), suggesting a video viewing pattern similar to that of the amalgam group. There were no significant differences between the three groups in terms of sex, learning groups, class format preference, providing comments, total online time, and webpage counts.

The degrees of pre-class learning satisfaction differed between the amalgam and either fine groups (89% vs. 93%, *p* = 0.018). In the final assessment of learning outcomes after face-to-face class learning, students in the amalgam group scored higher in the essay question part compared to the other groups (amalgam vs. nanolearning, *p* = 0.027; all three comparisons, *p* = 0.057). There were no differences in scores in the clinical case analysis part.

Regarding the quantitative learning feedback for specific core concepts, there was neutral agreement with previous understanding for C3, C4, and C6. C4 and C6 were concepts that most students wanted to learn more about, while C1 was the concept that most students suggested deleting (especially expressed at a high rate in the separate group). Students showed improvements in their knowledge of C2, C3, C4, C5, and C6 (over 50%) (Supplementary Table S2). The distribution patterns for the themes of concept agreement, desire to learn more about a concept, suggesting deletion of a concept in class, and improvement in understanding did not differ between the amalgam, nanolearning, and either fine groups, except for C1 and C2 (desire to learn more) and C6 (concept agreement) (Supplementary Table S2).

### Factors associated with preclass video format preference in multivariable analysis

A multiple general linear regression model was developed to analyze the factors influencing pre-class video format preference (represented by y values of 0, 1, and 2, corresponding to amalgam, either fine, and nanolearning formats, respectively). Factors associated with the preference of the nanolearning video format included being male, suggesting the deletion of specific concepts (C1, C2, and C4), and agreement with concept 4 (Table 2). On the other hand, factors associated with the preference of the amalgam microlearning video format included agreement with concept 6, a higher sum of concepts suggested for deletion, and higher scores in the essay question section (Table 2).

**Table 2:** Significant factors associated with preclass video format preference by multiple linear regression modeling with backward selection.

Variables	Coefficient (95% confidence interval)	*P*
Male	0.212 (0.012–0.683)	0.042
Perceived concept changing		
C4	0.218 (−0.005–0.344)	0.057
C6	−0.279 (−0.433– −0.044)	0.017
Further discussion of the concept in class is not needed		
C1	0.452 (0.291–1.044)	0.001
C2	0.250 (−0.010–0.941)	0.055
C4	0.385 (0.277–1.549)	0.005
Sum of chosen concepts	−0.400 (−0.747– −0.153)	0.003
Essay question score	−0.261 (−0.387– −0.038)	0.017

y = 0, 1, 2, representing format choices of amalgam, either fine, and nanolearning, respectively.

Parameters associated with video viewing pattern (completion percentage in nanolearning or amalgam microlearning videos and webpage counts) were not included in selection process.

C, core concept^#^.

# Core concepts of the ‘acute liver failure’ course (24).

C1. Acute liver failure is a systemic syndrome.

C2. The international normalized ratio of prothrombin time is a diagnostic and prognostic factor of acute liver failure.

C4. Renal dysfunction due to hepatorenal syndrome is much less frequently observed than that due to dehydration, infection, or drug toxicity.

C6. Macrophage plays a vital role in body fluid status dynamic fluctuation during the disease course.

### Student feedback

Table 3 provides student feedback following preclass online video learning. It includes examples of full-length comments from individual students with multiple codes. Across the three groups, students utilized this platform to express their perspectives on the learning experiences, which involved watching videos, asking specific questions, and showing appreciation. It can be inferred that the length of the videos, whether amalgam or nanolearning, was perceived as short enough and convenient for learning. For instance, students who voted for the amalgam microlearning video format commented, “I appreciated the teacher’s ability to produce short videos instead of long, full-length ones” and “these short videos are clear and comprehensive.” Some students explained their preferences in their comments. For example, one student stated, “I do not know where to start with so many items” (*amalgam*), while another expressed, “Separate videos help me clarify concepts individually, and along with the titles, allow me to quickly grasp the key points and learn” (*nanolearning*). Students who chose the “either fine” option expressed their appreciation for the teacher’s ability to crystallize key points within a clear framework (Table 3). In summary, students in all three groups utilized the platform to share their learning experiences, ask questions, and express appreciation. The length of videos, whether amalgam or nanolearning, was considered short and convenient for learning.

**Table 3:** Students’ feedback, coding, and examples.

Coding category*	Code number		
Number of participants	Amalgam	Nanolearning	Either fine
(*n* = 87)	(*n* = 52)	(*n* = 10)	(*n* = 25)
General: expressing gratitude, had learnt a lot, terrific, or effort appreciation	32	7	20
*Example*	Great thanks for offering these important concepts. I believe that these are teacher’s crystalized essence to clarify misunderstanding through the years of teaching. They are more like a “level-up” manual. Those videos are short but understandable. I do lean a lot and I hope the teacher can add more concepts in the future *(general, learning experiences, video) (either fine).*		
Specific: learning content query and class content request	16	2	5
*Example*	The short and conceptual preclass video learning is great in terms of time-saving, topics understanding, and appealing learning interests. In supplementary learning materials, it is said that protein-restriction is not necessary in acute liver failure and branch-chain amino acids are better than amino acids of aromatic rings. Why and how to apply clinically? *(learning experiences, video, general, query) (amalgam).*		
Preclass learning experience sharing, course design & framework	16	2	8
*Example*	The whole preclass learning process is very interesting. Moreover, the entry points are not overlapped with previous learning. The learning objectives include pathogenesis, clinical management, and prognosis judgment. All these bring me a brand-new understanding of acute liver failure *(learning experiences) (either fine).*		
Video specification	13	1	4
*Example*	Separated videos are very helpful for clarifying concepts, particularly emphasized with individual titles. Through these I can quickly catch the learning points and get learned a lot. I really appreciate the teacher’s arrangement and thank you *(video, learning experiences, general) (nanolearning).*		
Miscellaneous	2	0	1
*Example*	Learning materials are concise and convey concepts clearly. Thank you for the elaborative and self-explaining videos. We get to know more of the topics which is thought-provoking and facilitates discussion to each other. The color of some words in slides is close to that of the background and does not have enough contrast (*learning experiences, video, general, others*) (*amalgam*).		

* Examples of remarks and composite codes.

## Discussion

### Microlearning video vs. nanolearning video: how short is short enough?

In our study, most students appreciated the design of short videos into preclass blended learning and each student had his/her own favor. Microlearning video was preferred in over half of students. The ideal length of the videos one creates depends on the content, context, and viewer (29). However, it’s important to consider that viewers typically have short attention spans and numerous distractions (29). Wistia summarized the relationship between average viewer engagement and video length in a heatmap and suggested that engagement starts to decline sharply after the 2 min mark, with a noticeable drop from approximately 70% to slightly above 50% between the 6 and 7 min marks. After this point, engagement tends to stabilize, showing a slower decline until the video length reaches 12 min, where it once again experiences a rapid drop (29). However, if the video content per se can raise interest or curiosity, the factor of video length might act as a spark plug to ignite further learning activities (25). We can suggest that viewer engagement significantly decreases between 2 and 3 min. If the content requires more time to convey the message effectively, aim for videos ranging from 6 to 12 min in length (30). When unsure, it’s advisable to make the content shorter (29). Our videos fall into the category of explainer videos, which are generally animated and concise, serving as trailers for businesses (31). The length should not exceed 60 to 90 s, and shorter videos are generally more effective (31). Returning to our study, students’ preference for preclass video formats could be attributed to the content and context (specifically, self-learning reflection of several concepts) as well as the viewers (in this case, students’ short-term outcomes). Nonetheless, these formats of learning are not yet intended to replace a complete course in a subject (32) but in the context of blended learning of medical education, such as in fitting in our preclass learning modules together with applying strategy utilizing threshold concepts, can accommodate helpful concepts and information in a short period of time and facilitate learning in the later phase (1, 10, 25). Further study is needed to validate our findings and interpretation externally to other disciplines or learning topics.

### Balance between piecemeal learning and integral learning

The amalgam group performed better in the essay question but spend less total time and counts in online learning part of blended medical education. Although nanolearning is effective for conveying individual concepts in extremely short but memorable bursts (11), it remains concerned that integrated learning might be lacking through many piecemeal bite-sized learning processes. To achieve the benefit of nano- or micro-learning efficiency, for educational videos with long video length, video chapterization help viewers navigate the video and allow for a better understanding of what to expect in the video (33). Chapterization, which is now extremely widespread in social media (33), may essentially remove the need for an either/or dichotomy as it basically combines both options. In addition, there is data to show it improves social media engagement (33). However, if a student chose selective chapters to learn, they still risk losing a chance of integrative learning. Microlearning credentialing could safeguard against the risk (9). Besides, it would be revolutionary if integrative, in-depth learning could also be accomplished in the nanolearning frame.

Students in the either fine group reported higher preclass learning satisfaction levels but their online records showed a similar pattern in the amalgam group. They probably explored nanolearning videos first then shifted to the amalgam video (Table 1). This group (the second largest in our study) may be flexible learners with learning resilience, and provided feedback with high degrees of satisfaction. While restricted learning pattern may limit the learning dimension and degrees students could achieve in Millennial and Gen Z leaners, multiple and diverse learning strategies could potentially unleash the learning boundary and barriers. Blended medical education is a platform to achieve that under appropriate instructional design and content re-arrangement. A continuous refining process may be needed to meet the need across heterogeneous groups, school years, and generations.

### Applications of operationalization in other disciplines

In other disciplines, nanolearning and microlearning have been defined and applied in ways that support our study’s framework. Nanolearning is commonly operationalized as ultra-short, focused content—such as one-minute videos or quick reference tips—designed to deliver a single concept or skill efficiently (9). This format is widely used in fast-paced fields like corporate training and healthcare, where time-sensitive, just-in-time learning is essential (4, 6). Microlearning, by contrast, often involves slightly longer modules—typically around five to ten minutes—that cover multiple related concepts within a single session (14). In language learning and Science, Technology, Engineering, and Mathematics (STEM) education, for instance, microlearning supports conceptual integration and builds broader understanding within compact time frames (34). By adopting these conceptual definitions of nanolearning and microlearning, our study aligns with current trends toward flexible learning modalities and offers potential for broader application across disciplines.

### Specific recommendations for educators on how to design and integrate nanolearning/microlearning videos

When designing nanolearning and microlearning videos, educators may consider starting with clear, focused objectives—covering one concept in nanolearning and grouping related ideas in microlearning. Using simple visuals and friendly, concise language can help make the content more accessible. Keeping a steady pace, with a quicker flow for nanolearning and gentle transitions for microlearning, supports better understanding. Including short reflection prompts or self-check questions can also encourage active learning. These videos work well when designed as flexible, modular tools and can be improved over time with learner feedback and usage insights.

## Limitations

This study is grounded in the post-positivist research paradigm, which acknowledges that while objective reality exists, it can only be understood imperfectly due to inherent limitations in human observation and interpretation. However, the paradigm here supports a systematic and empirical approach to inquiry, emphasizing critical evaluation, multiple data sources, and contextually embedded interpretations to enhance the credibility and validity of findings. The definition of nanolearning and microlearning in this study is arbitrary categorized based on video length and amalgam nature, which may not be appropriate in broader terms as the learning content can be either text, picture, or simply sound. The study results based on the framework of preclass video learning in blended medical education may limit the external application of instructional design for a traditional class. The study lacked appropriate control, as students were grouped according to their preferences and observed throughout the learning journey, but they were not randomized into groups. Although the nanolearning videos had been used for years in our previous studies (10, 25), the course development and summative assessment in this study require instructional design models [e.g., the ADDIE (Analysis, Design, Development, Implementation, Evaluation) model] (1) for solid validation, which will inform future studies. The sample size of the nanolearning group might not be large enough to show statistical significance, if any. Single open-ended question may limit the validity for qualitative analysis as it is not strategically developed and we cannot approach about why some participants did not provide any comments on the questionnaire. However, it offers respondents an opportunity to voice their opinion and to ask for clarification or information about the learning (35), which plays a major part in practicing precision medical education. Because the subsequent analysis complemented quantitative analysis (including other questionnaires, online records, and assessment scores) each other and altogether reached a robust interpretation, the potential bias, if ever there, would be less.

## Conclusion and further work

Although students had individual preferences for nano- or micro-learning video format, they all found short videos to be enjoyable as pre-class learning modules. Educators are encouraged to choose and condense the most essential content in one bite-sized information snippet for nano- or micro- learning. This strategy can appeal to young generations, facilitate engagement, and save learning time. By incorporating this strategic approach into blended learning, a synergistic spark could be ignited to enhance the learning efficiency and knowledge retention in blended medical education.

Students in the either fine group reported higher preclass learning satisfaction levels while those in the amalgam group performed better in the essay question part of the final assessment. How can we make medical education work well and improve student satisfaction? Given that Generation Z has grown up in the digital era, the future generations would anticipate a transformation in the way knowledge is acquired, making the learning experience smoothly and even hardly recognizable (36). As multitaskers and technology savvy, they have shorter learning attention spans and prefer active learning modes (37). To make medical education work well, more attractive, and more effective, we have to respond to their preferences (38). Looking forward, nano- or microlearning can be part of precision medical education that help address the needs across the future generations (1).

## Data Availability

The raw data supporting the conclusions of this article will be made available by the authors, without undue reservation.
