# Supplementary material for: Preclass video nanolearning or microlearning in blended medical education
Source: Front Med (Lausanne). 2025 Oct 7;12:1639475. doi: 10.3389/fmed.2025.1639475 (PMC12538294; doi:10.3389/fmed.2025.1639475)

Supplementary Table S1. Online questionnaire form

Part I

Q1: Compared with my previous understanding, my knowledge of the core concept (C)# after watching the preclass self-learning online video is

C1：
- ☐ Totally changed
- ☐ Largely changed
- ☐ Half and half
- ☐ Mostly unchanged
- ☐ Totally unchanged

C2：
- ☐ Totally changed
- ☐ Largely changed
- ☐ Half and half
- ☐ Mostly unchanged
- ☐ Totally unchanged

C3：
- ☐ Totally changed
- ☐ Largely changed
- ☐ Half and half
- ☐ Mostly unchanged
- ☐ Totally unchanged

C4：
- ☐ Totally changed
- ☐ Largely changed
- ☐ Half and half
- ☐ Mostly unchanged
- ☐ Totally unchanged

C5：
- ☐ Totally changed
- ☐ Largely changed
- ☐ Half and half
- ☐ Mostly unchanged
- ☐ Totally unchanged

C6：
- ☐ Totally changed
- ☐ Largely changed
- ☐ Half and half
- ☐ Mostly unchanged
- ☐ Totally unchanged

Part II

Q2: Of which learning video(s) I learn most?

☐C1, ☐C2, ☐C3, ☐C4, ☐C5, ☐C6

Q3: I want to learn more about which concept(s)?

☐C1, ☐C2, ☐C3, ☐C4, ☐C5, ☐C6

Q4: I hope the teacher can have more discussions on the topic(s) of

☐C1, ☐C2, ☐C3, ☐C4, ☐C5, ☐C6

Q5: I hope the teacher can delete or decrease discussions on the topic(s) of

☐C1, ☐C2, ☐C3, ☐C4, ☐C5, ☐C6

Q6: If conditions permit, my preferred class format for the upcoming class is

☐Face-to-face

☐Online

☐Both

Q7: I feel _____ of the preclass video learning

☐Very dissatisfied, ☐Dissatisfied, ☐Neither satisfied nor dissatisfied, ☐ Satisfied, ☐Very satisfied

Q8: I prefer the video format for learning as an amalgam one, separate items (nanolearning), or either way fine

☐Amalgam one , ☐Separate items, ☐Either fine

Part III

My comment and/or questions____________________________________

#Core concepts of the 'acute liver failure' course[24]

C1. Acute liver failure is a systemic syndrome.

C2. The international normalized ratio of prothrombin time is a diagnostic and prognostic factor of acute liver failure.

C3. Blood urea nitrogen and phosphate are liver-related biomarkers in acute

liver failure.

C4. Renal dysfunction due to hepatorenal syndrome is much less frequently observed than that due to dehydration, infection, or drug toxicity.

C5. Deterioration of hepatic encephalopathy can be reversed by correcting trigger/aggravating factors—usually infection, bleeding, or dehydration.

C6. Macrophage plays a vital role in body fluid status dynamic fluctuation during the disease course.

Table S2. Quantitative learning feedback to specific core concepts (C)[#] after preclass video learning

	All	Amalgam	Nanolearning	Either fine	P	
n	140	79	18	43	Amalgam vs. Nanolearning	All 3
Perceived concept changing*	140	79	18	43		
C1	4 (4-4)	4 (4-4)	4 (4-4.25)	4 (3-4)	0.818	0.791
C2	4 (3-4)	4 (3-4)	3.5 (3-4)	3 (3-4)	0.883	0.864
C3	3 (2-4)	3 (2-4)	3 (3-3.25)	3 (2-4)	0.080	0.181
C4	3 (2-4)	3 (3-4)	3.5 (3-4)	3 (2-4)	0.141	0.176
C5	4 (3-4)	4 (3-4)	3.5 (3-4)	4 (3-4)	0.749	0.243
C6	3 (3-4)	3 (3-4)	3 (3-4)	3 (2-4)	0.197	0.036
Want to learn more	132	75	16	41		
C1	28	17	0	11	0.036	0.045
C2	38	18	2	18	0.508	0.026
C3	58	32	7	19	>0.999	0.967
C4	84	46	11	27	0.777	0.840
C5	70	37	10	23	0.414	0.556
C6	79	47	8	24	0.404	0.604
Sum of chosen concepts	3 (1-3)	3 (1-3)	2.5 (1-3.75)	3 (1-3.5)	0.652	0.548
Further discussion in class is not needed	91	55	13	23		
C1	54	30	11	13	0.061	0.137
C2	17	11	1	5	0.437	0.638
C3	9	7	0	2	0.331	0.545
C4	9	4	2	3	0.322	0.414
C5	12	11	0	1	0.107	0.073

C6	5	5	0	0	0.575	0.320
Sum of chosen concepts	1 (1-1)	1 (1-1)	1 (1-1)	1 (1-1)	0.587	0.770
Learnt most	136	78	18	40		
C1	43	26	5	12	0.784	0.868
C2	67	37	9	21	>0.999	0.912
C3	86	46	13	27	0.422	0.498
C4	83	47	13	23	0.425	0.561
C5	75	44	10	21	>0.999	0.940
C6	72	42	8	22	0.602	0.760
Sum of chosen concepts	3 (1-5)	3 (1.75-5)	2.5 (1-6)	3 (1-5)	0.969	0.991

*Five-point Likert scale: totally unchanged (1) to totally changed (5)

#Core concepts of the ‘acute liver failure’ course[24]

C1. Acute liver failure is a systemic syndrome.

C2. The international normalized ratio of prothrombin time is a diagnostic and prognostic factor of acute liver failure.

C3. Blood urea nitrogen and phosphate are liver-related biomarkers in acute liver failure.

C4. Renal dysfunction due to hepatorenal syndrome is much less frequently observed than that due to dehydration, infection, or drug toxicity.

C5. Deterioration of hepatic encephalopathy can be reversed by correcting trigger/aggravating factors—usually infection, bleeding, or dehydration.

C6. Macrophage plays a vital role in body fluid status dynamic fluctuation during the disease course.

Supplementary Figure S1. Participants

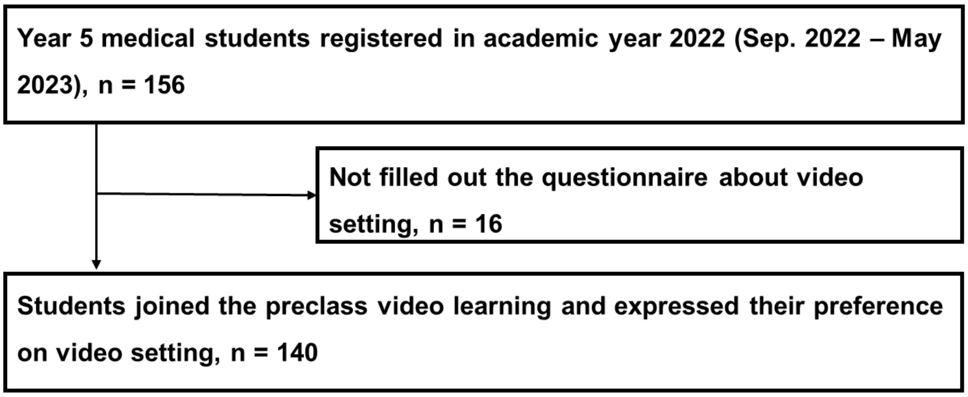

Supplement: Supplementary file 1 [file Presentation_1.pdf]
